# The Madeira Archipelago As a Significant Source of Marine-Derived Actinomycete Diversity with Anticancer and Antimicrobial Potential

**DOI:** 10.3389/fmicb.2016.01594

**Published:** 2016-10-07

**Authors:** Alejandra Prieto-Davó, Tiago Dias, Sofia E. Gomes, Sara Rodrigues, Yessica Parera-Valadez, Pedro M. Borralho, Florbela Pereira, Cecilia M. P. Rodrigues, Ilda Santos-Sanches, Susana P. Gaudêncio

**Affiliations:** ^1^Laboratorio de Productos Naturales Marinos, Facultad de Química, Universidad Nacional Autónoma de México, Unidad SisalSisal, Mexico; ^2^LAQV-REQUIMTE, Department of Chemistry, Faculty of Science and Technology, Universidade NOVA de LisboaCaparica, Portugal; ^3^UCIBIO-REQUIMTE, Department of Life Sciences, Faculty of Science and Technology, Universidade NOVA de LisboaCaparica, Portugal; ^4^Research Institute for Medicines (iMed.ULisboa), Faculty of Pharmacy, Universidade de LisboaLisbon, Portugal; ^5^Department of Biochemistry and Human Biology, Faculty of Pharmacy, Universidade de LisboaLisbon, Portugal

**Keywords:** marine actinomycetes, marine sediments, natural product discovery, Madeira Archipelago, anticancer activity, antimicrobial activity, marine actinomycete phylogeny, cultivable bacterial diversity

## Abstract

Marine-derived actinomycetes have demonstrated an ability to produce novel compounds with medically relevant biological activity. Studying the diversity and biogeographical patterns of marine actinomycetes offers an opportunity to identify genera that are under environmental pressures, which may drive adaptations that yield specific biosynthetic capabilities. The present study describes research efforts to explore regions of the Atlantic Ocean, specifically around the Madeira Archipelago, where knowledge of the indigenous actinomycete diversity is scarce. A total of 400 actinomycetes were isolated, sequenced, and screened for antimicrobial and anticancer activities. The three most abundant genera identified were *Streptomyces, Actinomadura*, and *Micromonospora*. Phylogenetic analyses of the marine OTUs isolated indicated that the Madeira Archipelago is a new source of actinomycetes adapted to life in the ocean. Phylogenetic differences between offshore (>100 m from shore) and nearshore (< 100 m from shore) populations illustrates the importance of sampling offshore in order to isolate new and diverse bacterial strains. Novel phylotypes from chemically rich marine actinomycete groups like MAR4 and the genus *Salinispora* were isolated. Anticancer and antimicrobial assays identified *Streptomyces, Micromonospora*, and *Salinispora* as the most biologically active genera. This study illustrates the importance of bioprospecting efforts at unexplored regions of the ocean to recover bacterial strains with the potential to produce novel and interesting chemistry.

## Introduction

Marine actinomycetes have proven to be an important source of novel secondary metabolites, as well as a promising source of pharmaceutically important agents (Magarvey et al., 2004; Jensen et al., 2005a,b; Lam, 2006; Prudhomme et al., 2008; Asolkar et al., 2009; Rahman et al., 2011; Subramani and Aalbersberg, 2012). The production of diverse secondary metabolites by marine-derived actinomycetes has been shown to be even greater than that of the most prolific natural source of bioactive secondary metabolites to date: the *Streptomyces* genus (Dharmaraj, 2010). For example, members of the marine-obligate genus *Salinispora*, which have proven to be a particularly rich source of novel chemical structures and novel classes of compounds (Jensen and Mafnas, 2006), including some in phase II cancer clinical trials (Cragg et al., 2014).

Different environmental pressures may drive bacteria to adapt to a certain location. These bacteria may harbor specific genes responsible for the synthesis of large multi-enzymatic compounds like polyketides and non-ribosomal peptide, or hybrids of both (Penn et al., 2009; Bose et al., 2014). This hypothesis is supported by the identification of 21 genomic islands found in the *Salinispora tropica* and *Salinispora arenicola* genomes, where species specific genes responsible for the synthesis of numerous natural products can be found (Penn et al., 2009). Furthermore, recent studies support the ecological divergence of two co-occurring and closely related *Salinispora* spp., providing evidence that they can evolve fundamentally different strategies to compete and thrive in marine sediments (Patin et al., 2016). Further evidence for the effect of biogeography and ecological adaptations on the diversity of microorganisms has been observed by the dispersal limitations that led to the establishment of endemic, microdiverse populations of *Prochlorococcus* (Martiny et al., 2009), as well as the fact that numerous bacteria show a limited distribution within specific habitat types (Nemergut et al., 2011). As such, the cultivation of novel bacterial species from distinct oceanic locations remains a productive approach to natural product discovery (Becerril-Espinosa et al., 2013). The actinomycete diversity of sediments collected in the Pacific Ocean and Caribbean Sea has been investigated (Jensen and Lauro, 2008; Solano et al., 2009; Prieto-Davó et al., 2013). Over 500 *Actinobacteria* cultivars have been isolated from Palau marine sediments (Gontang et al., 2007), and actinomycete genera isolated from Mariana Trench sediments include *Dermacoccus, Kocuria, Micromonospora, Streptomyces, Tsukammurella*, and *Williamsia* (Pathom-aree et al., 2006). However, the actinomycete diversity from Atlantic Ocean sediments is highly unexplored (Weyland, 1969; Walker and Colwell, 1975; Williams et al., 1999; Fiedler et al., 2005) and some isolation studies have resulted in the isolation of several genera, including *Nocardia, Micromonospora, Microbispora*, and *Streptomyces*, from sediments collected in the Chesapeake Bay and North Sea (Weyland, 1969, 1984; Takizawa et al., 1993). More recent studies revealed very high abundances of *Micromonospora* and *Streptomyces* at the Trondheim Fjord, Norway (Bredholt et al., 2008), while some cultivation dependent studies of sediments from New Brunswick, Canada revealed the presence of *Micromonospora, Nocardia, Nocardiopsis*, and *Pseudonocardia*, in addition to the genus *Streptomyces* (Duncan et al., 2014, 2015). Other oceanic locations, like deep sediments of the Macaronesia region which is influenced by Saharan debris flow, have been a good source of *Streptomyces* and *Rhodococcus* genera (Stach et al., 2003).

In this study, a cultivation dependent approach was used to cultivate diverse marine actinomycetes from the Madeira Archipelago. The main goals of this work were to investigate the bacterial diversity of an unexplored location in the Atlantic Ocean and to search for novel marine-derived actinomycetes with potential bioactivity. Results revealed considerable actinomycete diversity in this region of the Atlantic Ocean, including strains that could represent novel species or novel ecotypes belonging to previously described marine groups. Furthermore, our study confirms the pharmaceutical potential of marine-derived actinomycetes from unexplored regions of the ocean floor.

## Materials and methods

### Sample collection

Six hundred and sixty-two ocean sediment samples were collected in the Macaronesia Atlantic ecoregion, offshore the Madeira Archipelago, in the Southern reaches of Madeira (24 stations) and Porto Santo Islands (15 stations) and in the Western reach of Desertas Islands (6 stations) from June 4th to 17th 2012. The samples consisted of 662 sediments alternating from fine muds to small rocks and small pieces of dead coral. The shallow sediments were collected by scuba diving from depths of 1–20 m. The deep water sediments were collected using a modified, surface-deployed sediment sampler (Kahlsico, El Cajon, CA, model #214WA110) that does not need to be attached to a winch and reached depths of up to 1310 m. Each sample was transferred to a labeled sterile bag (Nasco Whirl-pack) and immediately stored on ice for transportation to shore. Long term storage was at −20°C.

### Isolation and cultivation methods

The 662 sediment samples were processed using the following drying method: sediment previously stored at −20°C for 1 month was dried overnight in a laminar flow hood and, when clumping occurred, ground lightly with an alcohol-sterilized mortar and pestle. An autoclaved plug (1–2 cm in diameter) was pressed onto the sediment and then repeatedly onto the surface of an agar plate in a clockwise direction creating a serial dilution effect. This method is designed to reduce the numbers of Gram-negative bacteria and to enrich for slow-growing, spore-forming actinomycetes. The samples were processed and inoculated as described above onto the surface of one to three of the following agar media. All media were prepared with 1 l of natural seawater and DI water in the proportion 75:25 and contained the anti-fungal agent cycloheximide and w (100 mg ml^−1^). Medium 1 (A1) 18 g of agar, 10 g of starch, 4 g of yeast extract, 2 g of peptone, medium 2 (1/2 A1) 18 g of agar, 5 g of starch, 2 g of yeast extract, 1 g of peptone and medium 3 (SWA) 18 g of agar.

### Actinomycete quantification and isolation

Inoculated Petri dishes were incubated at room temperature (*c.* 25–28°C) and monitored periodically over 6 months for actinomycete growth. Actinomycetes were quantified on each plate by eye. Actinomycetes were recognized by the presence of filamentous hyphae and/or by the formation of tough, leathery colonies that adhered to the agar surface. Thus, only mycelium-forming bacteria belonging to the order *Actinomycetales* were included in this study. Only 198 samples yielded mycelium-forming bacteria. For every plate that yielded actinomycete colonies, the total number of colonies observed was counted and representatives of all morphotypes were obtained in pure culture by repeated transfer from a single colony. Four hundred and twenty-one of these colonies were successively transferred onto new media until pure cultures were obtained. Four hundred and eight actinomycete strains were grow in liquid culture (medium 1 without agar) and cryopreserved at −80°C in 10% glycerol. The taxonomic classification was performed by 16S rRNA sequencing analysis and the isolated strains were tested for the requirement of seawater for growth (see below).

### Effects of seawater on growth

All 408 isolated actinomycetes were tested for the requirement of seawater for growth, i.e., marine obligate evaluation. This accomplished by replacing seawater with deionized water in the growth medium and observing effects on growth (Jensen et al., 2005b). Using a sterile loop, cells from a well-defined colony were removed from an A1 plate prepared with natural seawater and streaked onto plates of A1 prepared with seawater and deionized water. Plates were incubated at 25–28°C and growth was monitored for 6 months. The sea water obligate strains were replated three times for confirmation.

### DNA extraction, 16S rRNA gene amplification, and sequencing and taxonomic identification

All 421 pure strains were primarily grouped based on the presence or absence of aerial mycelium, on colony size, morphology, colony color, spore appearance, diffusible pigment production, and the presence or absence of aerial hyphae and the effects of seawater on growth. Representatives from each group were selected for 16S rRNA gene sequencing and phylogenetic analysis. Strains were cultured in 4 ml of medium A1, shaken at 200 rpm and 25°C for 3–7 days. The Wizard® Genomic DNA Purification Kit (Promega, Madison, WI, USA) from Gram positive bacteria protocol was used as described in the Wizard® Genomic DNA Purification Kit Technical Manual, #TM050, with minor modifications. We have found that longer incubation periods of the lytic enzyme (i.e., lisozyme) and the RNase solution were needed to obtain efficient amounts of genomic DNA. The 16S rRNA gene was polymerase chain reaction (PCR) amplified using the primers 27F (5′-AGAGTTTGATCCTGGCTCAG-3′) and 1492R (5′-TACGGCTACCTTGTTACGACTT-3′) (Gontang et al., 2007) and the products purified using SureClean PCR cleanup kit (BioLine, London, UK), using the protocol provided by the manufacturer. Purified PCR reactions were cycle-sequenced with the primers listed above at STAB VIDA, Lda (http://www.stabvida.net/), using ABI BigDye® Terminator v3.1 Cycle Sequencing Kit and purified. Purified products were run on an ABI PRISM® 3730xl Genetic Analyzer and sequence traces were edited using Sequencing Analysis 5.3.1 from Applied. All obtained sequences were compared to the GenBank database by the blastn algorithm.

### Nucleotide sequence accession numbers

All non-chimeric nucleotide sequences from the 400 cultivable actinomycete strains reported in this study have been deposited in GenBank under accession numbers KT446002–KT446401, KJ685362–KJ685387, and KP869059–KP869064 available at http://www.ncbi.nlm.nih.gov/genbank (Supplementary Table 1).

### Operational taxonomic units (OTUs) groupings

OTUs were created using 400 partial 16S rRNA sequences (~350 bp) from the cultivated actinomycetes at a 97% identity using the command *cluster.classic* in the program MOTHUR (http://www.mothur.org/) which compares the sequences by an “average neighbor” algorithm (Schloss et al., 2009). A representative sequence from each OTU was acquired using the *get.oturep* command in MOTHUR and almost full sequences (~1300 bp) for these 71 representatives were obtained. These representative sequences were compared to the GenBank database in order to determine their taxonomic affiliation as well as the percent identity shared with the 16S rRNA from their nearest reported sequence (neighbor).

### Rarefaction and diversity estimation analyses

Four hundred partial sequences were used for a rarefaction analysis and a diversity estimation using the non-parametric statistical estimator Chao1 with the command *rarefaction.single* from MOTHUR.

### Novel OTU determination

Representative strains were compared to type strains in the EZTaxon database for 16S rRNA sequence similarity. When sequences were < 98% similar to the nearest type strain, all strains in that OTU were then compared to the type strain in order to determine if the whole OTU could be considered novel or not. We used 98% similarity to run a more stringent analysis when deciding for novelty of the strains.

### Phylogenetic analysis

The 71 full sequences and their GenBank nearest neighbor sequences were aligned using ClustalX and the alignment used to create a Maximum Likelihood phylogenetic tree of 1279 bp using MEGA version 6.06 (http://www.megasoftware.net) with 1000 bootstraps (Tamura et al., 2013).

### UniFrac analysis

Isolated actinomycetes from the Madeira Archipelago were divided into shallow (≤ 100 m) and deep (>100 m) environments and a UniFrac analysis was performed in order to highlight any phylogenetic differences among these environments (Lozupone and Knight, 2005). At the same time, samples were separated according to the sediment characteristics and a UniFrac dissimilarity dendogram was created. UniFrac uses presence and abundance information from OTUs combined with a phylogenetic tree in order to perform these comparisons. All analyses performed on UniFrac were weighted and normalized for a more robust result.

### Bacteria crude extracts preparation and fractionation

The total isolated 400 actinomycete strains were cultured under identical growth conditions. These consisted in growing each strain in 90 ml of medium M1 with shaking at 220 rpm at 25°C for 7 days. Seed cultures (5 ml in 50 ml flasks, medium 1) were grown for 7 days prior to transfer. The culture broth was extracted with EtOAc. The organic layer was concentrated to dryness in vacuum, and the residue re-suspended in DMSO at 10 mg ml^−1^ concentration.

### Anticancer screening in HCT116 human colon carcinoma cells

#### Cell culture

The HCT116 human colon carcinoma cell line was grown in McCoy's 5A supplemented with 10% fetal bovine serum, and 1% antibiotic/antimycotic (Invitrogen, Grand Island, NY, USA) and cultured at 37°C in a humidified atmosphere of 5% CO_2_ Cells were seeded in 96-well plates at 3750 cells/well for MTS metabolism assay.

### Crude extract and 5-FU exposure

Stock solutions of 10 mg ml^−1^ of actinomycete crude extracts and positive control 5-fluorouracil (5-FU) at 8 mM (Sigma) were prepared in dimethyl sulfoxide (DMSO). Twenty-four hours after cell platting, cells were exposed to serial dilutions of actinomycete crude extracts and 5-FU, or DMSO vehicle control, for 72 h. All test compounds, 5-FU or DMSO were serially diluted four-fold in culture medium.

### Evaluation of cytotoxicity

In order to determine cancer cell's response to chemotherapeutics and other compounds in targeted screenings, as well as to explore colon cancer signaling pathways, we tested the anticancer activity of actinomycete crude extracts in HCT116 cells. Activity was evaluated after 72 h of cell exposure to crude extract, and in parallel to the positive and vehicle controls. Anticancer activity was evaluated by CellTiter 96® Aqueous Non-Radioactive Cell Proliferation Assay (Promega, Madison, WI, USA), using 4-(4,5-dimethylathiazol-2-yl)-5-(3-carboxymethoxyphenyl)-2-(4-sulfophenyl), inner salt (MTS), according to the manufacturer‘s instructions. In brief, this is a colorimetric method for determining the number of viable cells in proliferation, cytotoxicity or chemosensitivity assays. The CellTiter 96® Aqueous Assay is composed of solutions of MTS and an electron coupling reagent, phenazinemethosulfate (PMS). Anticancer activity of each actinomycete crude extract was determined by the decrease in cellular reduction of MTS into a formazan product that is soluble in tissue culture medium. The absorvance of the formazan product at 490 nm can be measured directly from the 96-well assay plates without additional processing, correlating with the number of living cells in culture. Concisely, 20 μl of MTS/PMS solution (19:1) was added directly to each well of a 96 well plate containing 150 μl of each actinomycete crude extract dilution in culture media, and the quantity of formazan product was measured after 1 h of incubation, using a Bio-Rad microplate reader Model 680 (Bio-Rad Hercules, CA, USA) at 490 nm. Maximum half inhibitory concentration (IC_50_) values were determined with GraphPad Prism (version 5 GraphPad Software, San Diego CA, USA).

### Crude extracts antimicrobial screening

The antimicrobial activity was evaluated for the 400 crude extracts by performing screenings against two of the most important antibiotic-resistant microorganisms that cause nosocomial infections, specifically methicillin-resistant *Staphylococcus aureus* COL (MRSA; Gill et al., 2005) and vancomycin-resistant (vanA phenotype) *Enterococcus faecium* EF82 (VREF; Mato et al., 1996), using Brain Heart Infusion (BHI) medium (DIFCO Laboratories, Detroit, USA, 1 l DI water). The screenings were performed in 96 well plates; each crude extract, previously concentrated at 10 mg ml^−1^ in DMSO, was added to a log-phase grown culture (OD_600_ nm = 0.04−0.06) to a 2.5% (v/v) final concentration. All samples were two-fold serially diluted five times, resulting in final concentrations of the tested compounds ranging from 250 to 7.81 μg ml^−1^. Further dilutions were tested, values down to 0.03 μg ml^−1^, if the 7.81 μg ml^−1^ concentration showed inhibitory activity. After 18 h of incubation at 37°C, minimal inhibitory concentrations were determined by visual inspection and spectrophotometric analysis. The active crude extracts were re-tested for result confirmation.

## Results

**Figure 1:**
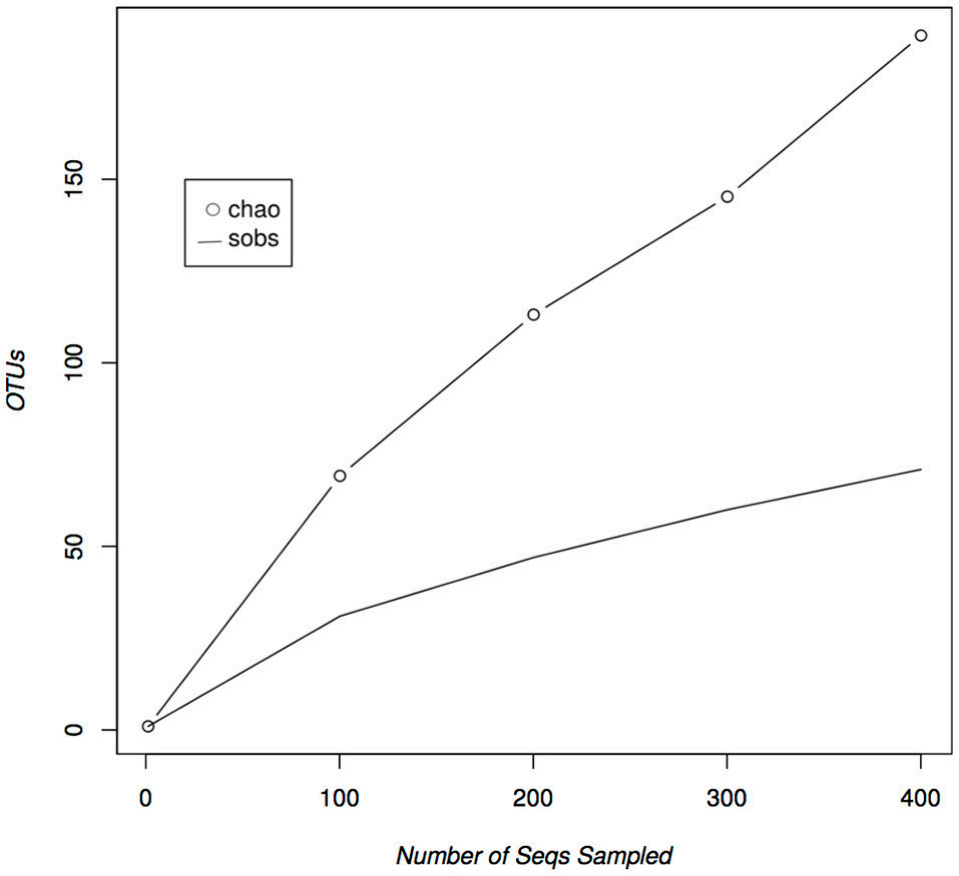
**Rarefaction curves of isolated marine derived actinomycetes from the Madeira Archipelago**. Both observed (sobs) and estimated (chao) diversity curves show that saturation has not been reached within the diversity of cultivated marine-derived actinomycetes.

Of the 421 isolated and sequenced strains, 408 were identified as actinomycetes according to a BLASTN search against the GenBank database. Eight of those sequences were suggested to be chimeras by the NCBI sequence submission process and were left out of our analyses. The remaining 400 sequences were used to calculate rarefaction and estimate the diversity of cultivable actinomycetes in the Madeira Archipelago. In total, the samples obtained from the Archipelago show that its sediments harbor at least 71 different Operational Taxonomic Units (OTUs) covering 24 actinomycete genera (Table 1). As in most cultivation studies, OTUs from the genus *Streptomyces* represented the great majority of the isolates. Almost the same number of *Micromonospora* OTUs, were recovered, but only 11 *Actinomadura* strains were isolated as compared to 90 from *Micromonospora*, demonstrating a redundancy in the strain diversity from the latter genus. Most of the other genera identified were represented by only one or two OTUs, even when the number of strains isolated was high (Table 1). This is likely due to the use of a conservative cutoff value to define distinct OTUs (97% sequence identity) each of which may encompass more than one species. For example, the genus *Salinispora* is represented by only one OTU, even though we know that two *Salinispora* species (*Salinispora pacifica* and *S. arenicola)* were isolated from the collected sediment. Nevertheless, even with this conservative grouping, rarefaction calculations of the cultivable diversity clearly show that the OTU richness asymptote (saturation) has not been reached (Figure 1). These results suggest that further sampling and the use of more isolation methods are likely to result in the isolation of additional cultivable actinomycete OTUs from the Archipelago sediment. A statistical estimation of cultivable richness using Chao1 also suggests that more actinomycetes OTUs, possibly an additional 118 OTUs, could be isolated from the collected sediment samples (Figure 1).

**Table 1:** **Name of genera and number of OTUs and strains for each genus isolated from the Madeira Archipelago marine sediments**.

**Genus**	**Number of OTUs**	**Number of strains**
*Streptomyces*	25	178
*Actinomadura*	8	11
*Micromonospora*	6	90
*Actinomycetospora*	5	10
*Pseudonocardia*	3	6
*Nocardiopsis*	2	28
*Stackebrandtia*	2	4
*Nonomuraea*	2	3
*Saccharomonospora*	2	2
*Mycobacterium*	2	2
*Salinispora*	1	34
*Verrucosispora*	1	15
*Nocardia*	1	4
*Actinocorallia*	1	2
*Gordonia*	1	2
*Jiangella*	1	1
*Rhodococcus*	1	1
*Millisia*	1	1
*Brevibacterium*	1	1
*Corynebacterium*	1	1
*Actinoalloteichus*	1	1
*Micrococcus*	1	1
*Microbacterium*	1	1
*Saccharopolyspora*	1	1

**Figure 2:**
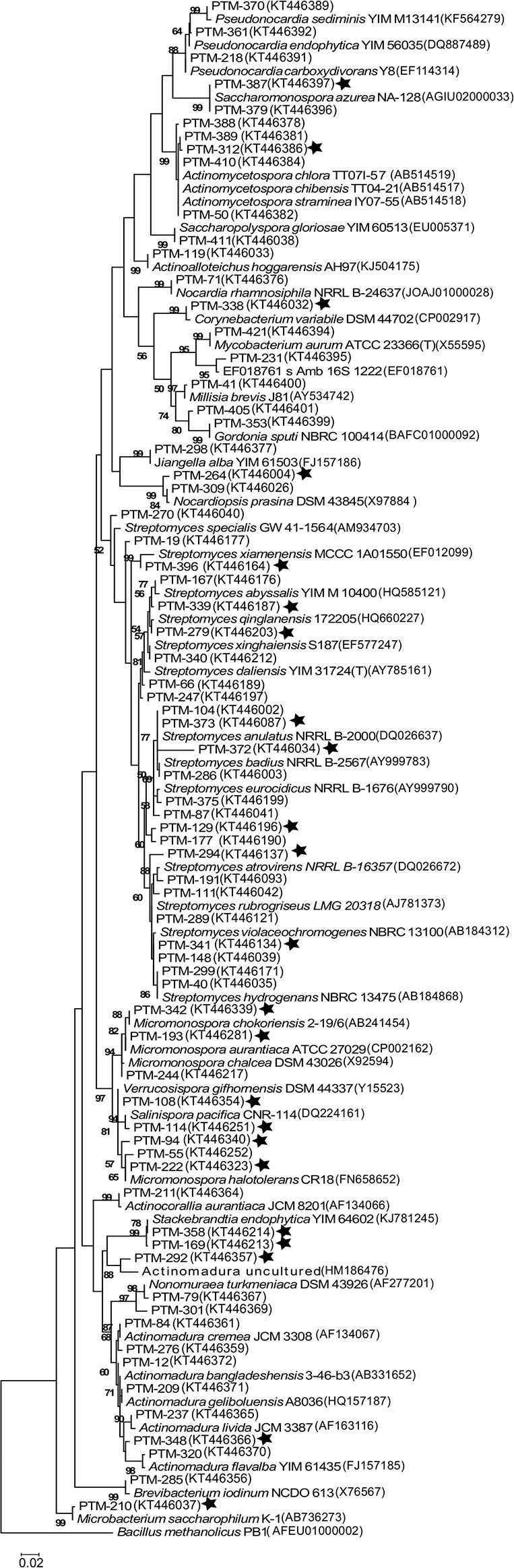
**Maximum Likelihood phylogenetic tree of the 16s rRNA gene from 71 representative actinomycete OTUs from the Madeira Archipelago and their GenBank nearest neighbor**. The tree was created using 1279 bp and 1000 bootstraps. Numbers in parenthesis are GenBank accession numbers and PTM number refers to internal reference collection number. Stars show those OTUs where strains with a requirement of seawater for growth were observed. *Bacillus methanolicus* was used as an outgroup.

In order to understand the evolutionary relationships among the OTUs isolated, a phylogenetic tree was constructed (Figure 2). The tree illustrates that most of the OTUs are highly similar to previously reported actinomycete sequences available in GenBank, although we did detect OTUs in the *Streptomyces, Stackebrandtia*, and A*ctinomycetospora* genera that are phylogenetically distinct. Furthermore, when the seawater requirement for growth is included in the tree (stars on Figure 2), we observe that this is not an exclusive trait of already described marine obligate genera (i.e., *Salinispora*). Rather, the requirement of seawater for growth is a widespread characteristic among isolates from many OTUs, suggesting that marine-derived actinomycetes from diverse genera are adapted to marine conditions.

**Figure 3:**
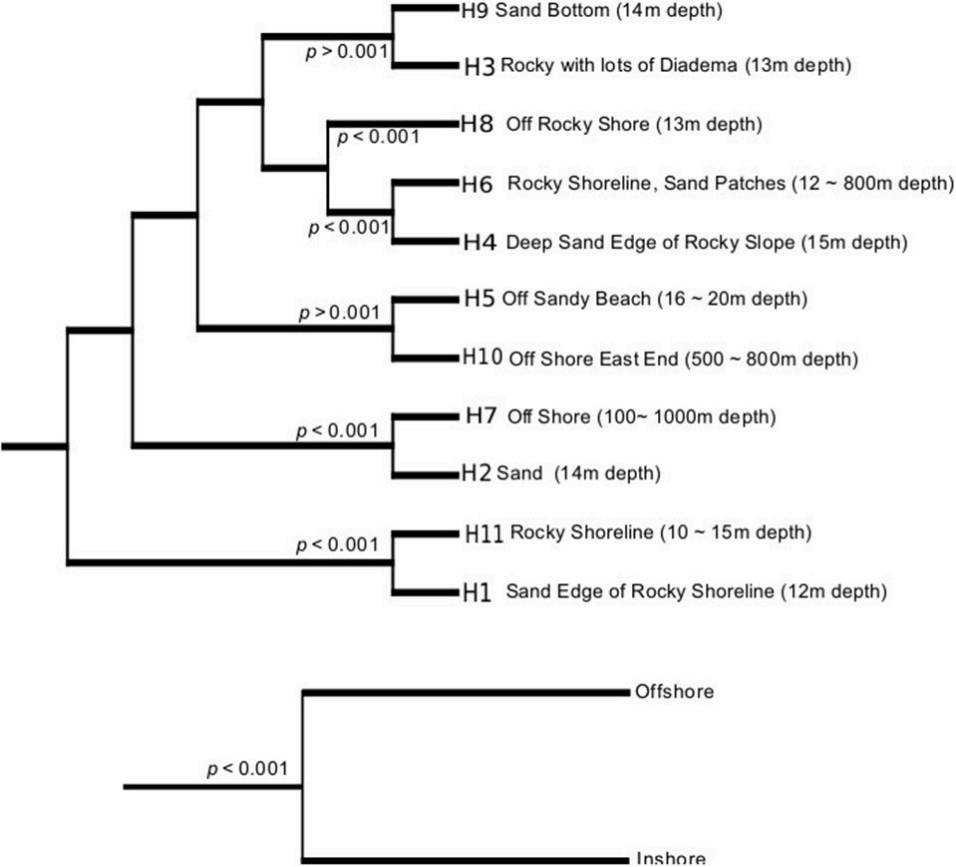
**UniFrac dissimilarity dendograms per habitat (top) and as a group of inshore and offshore samples (bottom)**.

In order to prioritize future sampling locations to improve bioprospecting efforts, we aimed to identify locations that yielded the most diverse groups of actinomycetes. In order to do so, UniFrac analyses were employed to examine actinomycete populations in two ways: First, we decided to weigh up the influence of land derived actinomycetes by comparing the diversity between inshore (≤ 100 m from shore) vs. offshore locations (>100 m from shore) and secondly, we wanted to examine the influence of the habitats' distinct sediment characteristics on the isolated actinomycete diversity. With these groupings, UniFrac dissimilarity dendograms were created (Figure 3). The results indicate that inshore and offshore samples yield statistically significant different actinomycete populations, indicating that future collections should aim to collect deep sediment samples far from shore in order to recover actinomycete strains different from those recovered from coastal areas. On the other hand, when the analyses were performed on representative habitats, the 11 habitats formed four different clades (Figure 3). Except for habitats “rocky shoreline” and “sand,” no significant phylogenetic differences among cultivable actinomycetes from different sediment types were encountered, suggesting that sediment type is not a characteristic that determines or influences the phylogenetic diversity of the actinomycetes cultivated with our methods. When rarefaction curves were applied to analyze the sampling effort for each habitat, we observed that all habitats were either undersampled or that the methodology applied did not allow for a wide diversity of actinomycetes to be recovered (Supplementary Figure 1). Shannon diversity index calculations revealed that, based on our cultivation strategies, the most diverse habitats were those with the highest number of isolates, (Table 2), suggesting that, in Madeira's sediments, further isolation techniques might yield additional cultivable actinomycete diversity. Further supporting this result is the fact that from 662 processed marine sediment samples only 198 returned actinomycetes.

**Table 2:** **Diversity and distribution of actinomycetes in the different habitats sampled from the Madeira Archipelago**.

	**H1**	**H2**	**H3**	**H4**	**H5**	**H6**	**H7**	**H8**	**H9**	**H10**	**H11**
OTUs	10	12	8	32	6	15	14	11	7	6	32
No. of Strains	14	28	14	104	7	39	33	20	13	11	117
No. of Samples	11	16	10	30	4	29	10	14	10	8	56
Strains/Sample	1.2	1.7	1.4	3.46	1.7	1.3	3.3	1.4	1.3	1.3	2.0
Shannon index	2.341	2.174	1.905	2.862	1.747	2.351	2.277	2.194	1.844	1.54	2.688

**Figure 4:**
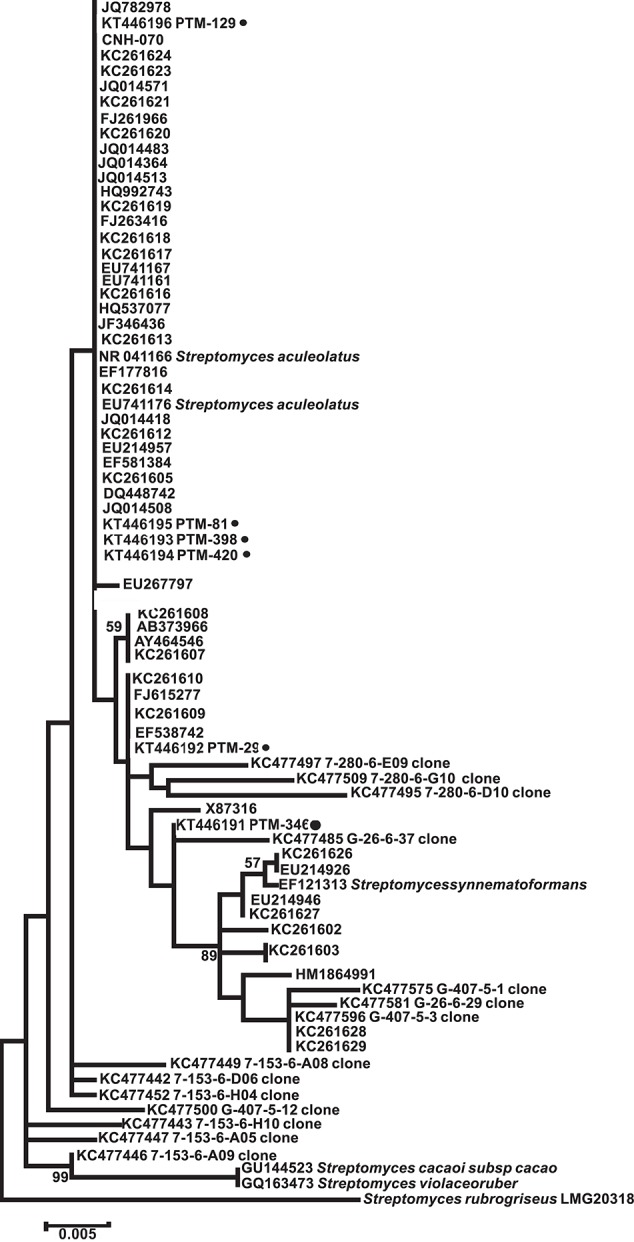
**Maximum Likelihood phylogenetic tree using 16s rRNA (1279 bp and 1000 bootstraps) from Madeira actinomycete strains belonging to the previously described MAR4 group**. Reference MAR4 strains were used to create the tree. PTM numbers of the Madeira strains are shown followed by a dark filled circle. *Streptomyces rubreogriseus* was used as an outgroup.

**Figure 5:**
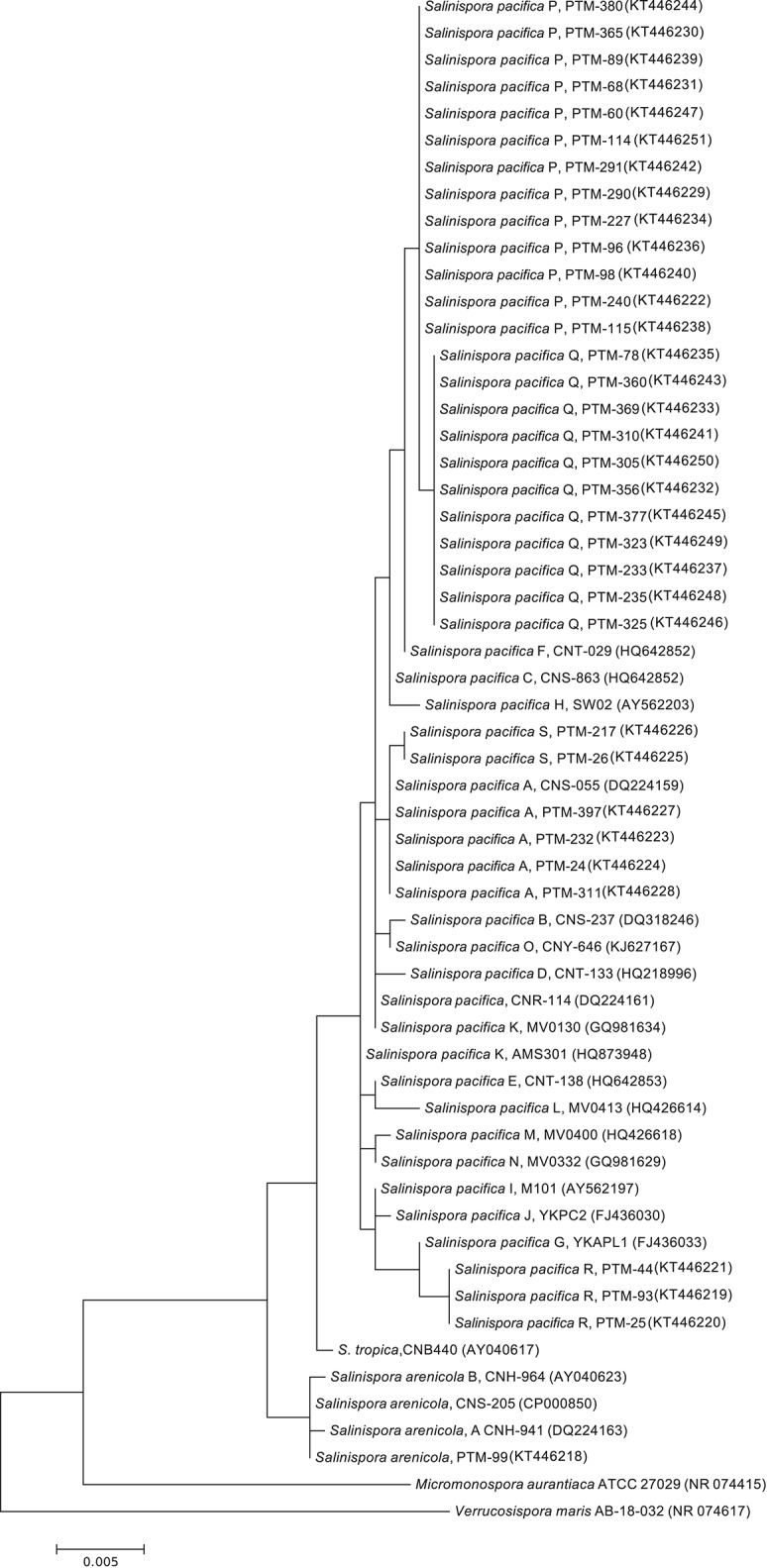
**Maximum Likelihood phylogenetic tree using 16s rRNA (1279 bp and 1000 bootstraps) from Madeira actinomycete strains belonging to the previously described *Salinispora* genus**. Reference *Salinispora* strains were used to create the tree. *Salinispora* species are named and followed by their GenBank accession number in parenthesis. PTM numbers of the Madeira strains are shown. *Verrucosispora maris* was used as an outgroup.

Because of our interest in the chemical capabilities of these microorganisms, a deeper exploration of two OTUs comprising the MAR4 and MAR1 (*Salinispora*) clades was performed. Our results showed six strains from the Archipelago that were distributed widely among previously described MAR4 diversity clades (Gallagher et al., 2013; Figure 4), with the possibility that some could be considered novel MAR4 phylotypes. Meanwhile, the phylogenetic analysis of *Salinispora* strains showed the presence of *S. pacifica* and *S. arenicola* strains in the Atlantic. Among these, four new phylotypes of *S. pacifica* were discovered (S, P, Q, and R, Figure 5), suggesting that bioprospecting efforts still play an important role in the discovery of novel phylotypes of chemically important marine actinomycete strains.

In order to gain insight into the bioactive potential of marine derived actinomycetes from the Madeira Archipelago, crude extracts from all 400 actinomycete were screened for anticancer activity in the HCT116 human colon carcinoma cell line and for antimicrobial activity against methicillin-resistant *Staphylococcus aureus* (MRSA) and vancomycin-resistant *Enterococcus faecium* (VREF) pathogens. From these, three biologically active actinomycete genera could be identified: *Streptomyces, Micromonospora, Salinispora* (Tables 3, 4). As a first look into the pharmaceutical application of these strains, bioactivity of crude extracts was measured. Bioactivity ranged from 0.65 to 59.05 μg ml^−1^ for HCT116 cell lines and ≤ 0.03–250 μg ml^−1^ for MRSA and VREF antimicrobial activity. The most bioactive strains belonged to the genus *Streptomyces*, and members from the marine clade MAR4 were bioactive in all tests. The *S. arenicola* isolate showed anticancer activity (as expected) and antimicrobial activity against MRSA at 62 μg ml^−1^ (Table 4). Surprisingly, none of the *S. pacifica* isolates showed biological activity in any of the bioassays, illustrating that the same species isolated from different locations in the ocean can vary in their secondary metabolite profiles or that cultivation techniques need to be optimized for compound production.

**Table 3:** **Antimicrobial results for marine actinomycetes crude extracts against methicillin-resistant *Staphylococcus aureus* COL (MRSA) and vancomycin-resistant (vanA phenotype) *Enterococcus faecium* EF82 (VREF)**.

**Strain/Extract code**	**GenBanK accession number**	**Strain description**	**MRSA bioassay (μg ml^−1^) MIC**	**VREF bioassay MIC**	**Archipelago location**	**Habitat**	**GenBanK accession number**
PTM-29	KT446192	*Streptomyces aculeolatus* MAR4	125	15.63	Madeira	H7	KP869059.1
PTM-81	KT446195	*Streptomyces aculeolatus* MAR4	1.95	≤ 0.03	Madeira	H1	KP869061.1
PTM-99	KT446218	*Salinispora arenicola* MAR1	62.50	–	Desertas	H11	KJ685387.1
PTM-105	KT446303	*Micromonospora aurantiaca* ATCC 27029	62.50	62.5	Porto Santo	H11	HG526643.1
PTM-128	KT446196	*Micromonospora aurantiaca* ATCC 27029	7.81	≤ 0.03	Madeira	H4	HG526643.1
PTM-289	KT446121	*Streptomyces rubrogriseus LMG 20318*	–	125	Madeira	H8	AJ781373.1
PTM-346	KT446191	*Streptomyces aculeolatus* MAR4	125	3.91	Madeira	H2	KP869060.1
PTM-384	KT446295	*Micromonospora aurantiaca* ATCC 27029	250	–	Madeira	H4	HG526643.1
PTM-398	KT446193	*Streptomyces aculeolatus* MAR4	–	31.25	Madeira	H7	KP869063.1
PTM-420	KT446194	*Streptomyces aculeolatus* MAR4	7.81	3.91	Desertas	H11	KP869064.1

–*, Not active; MIC, Minimum Inhibition Concentration.*

**Table 4:** **Cytotoxic activity (IC_50_ μg ml^−1^) of the marine actinomycetes crude extracts against HCT116 human colon carcinoma cells for 72 h**.

**Strain/Extract code**	**GenBank accession number**	**Strain description**	**HCT116 bioassay IC^a^_50_ (μg ml^−1^)**	**Archipelago location**	**Habitat**	**GenBank accession number**
PTM-15	KT446086	*Streptomyces anulatus* NRRL B2000	6.00	Desertas	H11	DQ026637.1
PTM-19	KT446177	*Streptomyces carpaticus* NBRC 15390	8.68	Madeira	H2	NR_112450.1
PTM-34	KT446147	*Streptomyces hydrogenans* NBRC 13475	9.70	Madeira	H8	AB184868.1
PTM-36	KT446065	*Streptomyces anulatus* NRRL B2000	59.05	Desertas	H11	DQ026637.1
PTM-46	KT446118	*Streptomyces rubrogriseus* LMG 20318	1.32	Desertas	H11	AJ781373.1
PTM-63	KT446126	*Streptomyces violaceochromogenes* NBRC 13100	13.99	Desertas	H11	AB184312.1
PTM-81	KT446195	*Streptomyces aculeolatus* MAR4	19.55	Madeira	H1	KP869061.1
PTM-85	KT446127	*Streptomyces violaceochromogenes* NBRC 13100	32.05	Desertas	H11	AB184312.1
PTM-99	KT446218	*Salinispora arenicola* MAR1	4.94	Desertas	H11	KJ685387.1
PTM-105	KT446303	*Micromonospora aurantiaca* ATCC 27029	7.90	Porto Santo	H11	HG526643.1
PTM-106	KT446085	*Streptomyces anulatus* NRRL B2000	9.98	Desertas	H11	DQ026637.1
PTM-126	KT446128	*Streptomyces violaceochromogenes* NBRC 13100	18.02	Desertas	H11	AB184312.1
PTM-128	KT446196	*Micromonospora aurantiaca* ATCC 27029	40.22	Madeira	H4	HG526643.1
PTM-304	KT446075	*Streptomyces anulatus* NRRL B2000	0.65	Desertas	H11	DQ026637.1
PTM-366	KT446072	*Streptomyces anulatus* NRRL B2000	24.98	Desertas	H11	DQ026637.1
PTM-392	KT446070	*Streptomyces anulatus* NRRL B2000	8.50	Desertas	H11	DQ026637.1
5-Fluorouracil		NA	0.19	NA	NA	NA
Oxaliplatin		NA	0.18	NA	NA	NA

a Cell viability was assessed using the MTS assay; NA, Not applicable.

## Discussion

The Portuguese archipelago Madeira is located in the Macaronesia Atlantic region, which emerges from the African tectonic plate and is found at the southern end of the Tore-Madeira ridge. The region has a unique biogeography and biodiversity. Madeira's superficial oceanic currents are associated with the North Atlantic current system and are not created by the winds of the Azores anticyclone. The deep water mass is composed of water from the North Atlantic, South Atlantic, and the Mediterranean Sea (Emery and Meincke, 1986; Cox, 1989). These unique characteristics, combined with the fact that there are no reports of cultivable actinomycetes from Atlantic Ocean sediments other than by Weyland (Weyland, 1969), make the Madeira Archipelago sediments a perfect target for marine sediment bioprospecting scientific studies.

Our bioprospecting study targeted cultivable actinomycete diversity that can be accessed using inexpensive and easy methods, including a sediment corer designed to be deployed from a small boat without the need to pay for expensive ship time.

The sediments proved to be actinomycete rich, yielding 400 cultivable actinomycetes from 198 marine sediments. A total of 24 genera, which included 71 different OTUs, were cultivated from sediments collected from various locations around the Madeira Archipelago. Furthermore, the estimated cultivable diversity calculations suggest these marine sediments harbor additional potential as a source of diverse actinomycetes. The majority of the cultivable diversity belonged to the genus *Streptomyces*, where the presence of 25 OTUs showed that the genus is still one of the most readily available for cultivation in marine sediments, even when recent studies have shown that other actinomycete genera can dominate marine sediment populations (Gontang et al., 2007; Claverías et al., 2015). These results most likely suggest that our cultivation methods were selective for this genus. Our sampling methods did result in isolation of 24 distinct genera, far beyond the actinomycete diversity cultivated from Atlantic marine sediments to date (Weyland, 1969, 1984; Stach et al., 2003; Maldonado et al., 2005; Duncan et al., 2014, 2015). Our 97% cutoff value for OTU grouping resulted in a conservative number of OTUs, even when the number of isolated strains was high (e.g., *Salinispora, Nocardiopsis)*. The use of a conservative cutoff value was not intended to define bacterial species. As such, we suggest there are more than 71 bacterial species represented by our 400 isolates. Because these OTUs shared >98% 16S rRNA gene sequence identity with strains already reported in public databases, we do not consider any of the isolates to be novel strains. Nevertheless, the requirement of seawater for growth suggests that some of the isolates could be novel species or novel ecotypes. There is evidence that the vast cultivable actinomycete diversity encountered in the Madeira sediments can be expanded since OTU saturation was not reached and the Chao1 diversity estimator suggests an additional 118 OTUs could be isolated from these sediments (Figure 1). In the future, cultivation efforts should be widened and more media or pretreatment methods should be used to access additional diversity (Janssen et al., 2002). Adaptations to the marine environment, as indicated by a requirement of seawater for growth, could be observed among most of the genera isolated in this study. Although only a few marine-obligate actinomycetes have been described (Maldonado et al., 2005; Tian et al., 2012), the high number of seawater-requiring strains suggests that the Madeira Archipelago is a source of actinomycetes adapted to life in the ocean.

A statistical difference between the cultivable actinomycete diversity observed in sediments close to the shore vs. offshore emphasizes the importance of collection trips that include the latter, remote locations. Moreover, distinct cultivable phylogenetic diversity was found in habitats composed of rocks and sand, although no other habitat from the remaining nine that were sampled indicated a strong relationship between the type of sediment and the cultivable actinomycete phylogenetic diversity present. This observation was also recorded by Duncan and collaborators who explored the distribution of actinomycetes in marine sediments from New Brunswick, Canada (Duncan et al., 2014). From the 11 habitats studied, the “deep sand end rocky slope” provided the most diverse assemblage of isolates (Table 2), and this higher relative cultivable diversity might be related to the fact that, on average, three strains were isolated per sample, while from the other habitats, the recovery of strains was lower than two per sample.

Although our study was aimed toward cultivable actinomycete diversity, we believe that the total actinomycete richness of these sediments is above that presented here. This can be assumed from two sources of information. First, our rarefaction curve did not reach saturation (Figure 1), suggesting the availability of more cultivable strains and second, recent cultivation-independent studies of marine-derived sediments have shown that 13% of the diversity in them belongs to the class *Actinobacteria* (Bienhold et al., 2016). Although the ease of access to this diversity has not been established, the intrinsic characteristics of the sediments can be used as a guide to design novel cultivation methods and bring these actinomycetes into laboratory culture. Studies like the one we present here show that the vastness of oceanic marine sediments allows for easily applicable sampling and cultivation methods to access a wide diversity of marine derived actinomcyetes with potential for natural product discovery.

One of the most important features of bioprospecting efforts, besides the discovery of novel cultivable phylogeny, is determining the bioactive potential of the isolated strains. By piecing these two components together, it is possible to isolate strains that belong to distinct marine groups of novel phylogeny and assess their capacity to produce interesting chemistry. Such is the case of actinomycetes belonging to the marine clade MAR4, as they have been reported to be an important source of hybrid isoprenoids (Gallagher et al., 2010) and a relationship between their phylogeny and the compounds they produce has been proposed (Gallagher et al., 2013). In this study, diverse MAR4 strains were isolated, and some showed interesting biological activities (see Tables 3, 4). Although none of the *S. pacifica* phylotypes were found to harbor bioactivity (vs. one *S. arenicola* with both antimicrobial and cytotoxic activities), their ability to produce novel chemistry and yield different biological activity has yet to be explored (Freel et al., 2011; Vidgen et al., 2012; Bose et al., 2014). Additionally, our sampling efforts led to the first report of *Salinispora* spp. from North Atlantic Ocean temperate waters. *Salinispora* spp. have only been isolated previously from warmer tropical waters (Jensen et al., 2015).

The bioactive potential of the 400 strains was assessed and 22 strains were found to have active crude extracts either as antimicrobial or as cytotoxic agents. It is interesting to highlight that, even though there were a total of 24 identifiable genera in our collection, only three showed bioactivities in our assays, suggesting that the study of marine *Streptomyces, Micromonopora*, and *Salinispora* is still an important and fruitful one with respect to discovering novel natural products.

Marine sediments from the Madeira Archipelago yielded a diverse assemblage of marine actinomycetes, the highest diversity yet seen in the Atlantic. Our bioprospecting study revealed that distinct populations of these microorganisms can be accessed in both near shore and offshore locations. Given the potential for bioactive compound production that marine actinomycetes have shown, both in the present and previous studies, we suggest that efforts to access offshore populations of actinomycetes should be undertaken and that more intensive cultivation methods be used to access the enormous diversity of potentially prolific chemical producers in the ocean.

## Author contributions

All authors listed, have made substantial, direct and intellectual contribution to the work, and approved it for publication.

## Funding

This work was supported by funding from FCT/MEC, through grants PTDC/QUIQUI/ 119116/2010, IF/00700/2014, UID/QUI/50006/2013 (LAQV) and UID/Multi/04378/2013 (UCIBIO) and co-financed by the ERDF under the PT2020 partnership agreement POCI-01-0145-FEDER - 007265 and POCI-01-0145-FEDER-007728. Funding from the EU 7th Framework Programme (FP7/2007–2013) under grant agreement PCOFUND-GA-2009-246542 and 269138-NanoGuard. YP acknowledges CONACYT for the M.Sc. fellowship grant 560614.

### Conflict of interest statement

The authors declare that the research was conducted in the absence of any commercial or financial relationships that could be construed as a potential conflict of interest. The reviewer SLL and handling Editor declared their shared affiliation and the handling Editor states that the process nevertheless met the standards of a fair and objective review.

## References

[B1] Asolkar R. N. Freel K. C. Jensen P. R. Fenical W. Kondratyuk T. P. Park E.-J.. (2009). Arenamides, A.-C., cytotoxic NF kappa B inhibitors from the marine actinomycete *Salinispora arenicola*. J. Nat. Prod. 72, 396–402. 10.1021/np800617a 19117399 PMC2837138

[B2] Becerril-Espinosa A. Freel K. C. Jensen P. R. Soria-Mercado I. E. (2013). Marine *Actinobacteria* from the Gulf of California: diversity, abundance and secondary metabolite biosynthetic potential. Antonie Van Leeuwenhoek 103, 809–819. 10.1007/s10482-012-9863-3 23229438 PMC4517938

[B3] Bienhold C. Zinger L. Boetius A. Ramette A. (2016). Diversity and biogeography of bathyal and abyssal seafloor bacteria. PLoS ONE 11:e0148016. 10.1371/journal.pone.0148016 26814838 PMC4731391

[B4] Bose U. Hewavitharana A. K. Vidgen M. E. Ng Y. K. Shaw P. N. Fuerst J. A.. (2014). Discovering the recondite secondary metabolome spectrum of *Salinispora* species: a study of inter-species diversity. PLoS ONE 9:e91488. 10.1371/journal.pone.0091488 24621594 PMC3951395

[B5] Bredholt H. Fjaervik E. Johnsen G. Zotchev S. B. (2008). Actinomycetes from sediments in the Trondheim Fjord, Norway: diversity and biological activity. Mar. Drugs 6, 12–24. 10.3390/md6010012 18648671 PMC2474955

[B6] Claverías F. P. Undabarrena A. Gonzalez M. Seeger M. Camara B. (2015). Culturable diversity and antimicrobial activity of *Actinobacteria* from marine sediments in Valparaiso bay, Chile. Front. Microbiol. 6:737. 10.3389/fmicb.2015.00737 26284034 PMC4516979

[B7] Cox M. D. (1989). An idealized model of the world ocean 1. The global-scale water masses. J. Phys. Oceanogr. 19, 1730–1752.

[B8] Cragg G. M. Grothaus P. G. Newman D. J. (2014). New horizons for old drugs and drug leads. J. Nat. Prod. 77, 703–723. 10.1021/np5000796 24499205

[B9] Dharmaraj S. (2010). Marine *Streptomyces* as a novel source of bioactive substances. World J. Microbiol. Biotechnol. 26, 2123–2139. 10.1007/s11274-010-0415-6

[B10] Duncan K. Haltli B. Gill K. A. Kerr R. G. (2014). Bioprospecting from marine sediments of New Brunswick, Canada: exploring the relationship between total bacterial diversity and *Actinobacteria* diversity. Mar. Drugs 12, 899–925. 10.3390/md12020899 24531187 PMC3944522

[B11] Duncan K. R. Haltli B. Gill K. A. Correa H. Berrué F. Kerr R. G. (2015). Exploring the diversity and metabolic potential of actinomycetes from temperate marine sediments from Newfoundland, Canada. J. Ind. Microbiol. Biotechnol. 42, 57–72. 10.1007/s10295-014-1529-x 25371290

[B12] Emery W. J. Meincke J. (1986). Global water masses - summary and review. Oceanol. Acta 9, 383–391.

[B13] Fiedler H. P. Bruntner C. Bull A. T. Ward A. C. Goodfellow M. Potterat O.. (2005). Marine actinomycetes as a source of novel secondary metabolites. Antonie Van Leeuwenhoek 87, 37–42. 10.1007/s10482-004-6538-8 15726289

[B14] Freel K. C. Nam S.-J. Fenical W. Jensen P. R. (2011). Evolution of secondary metabolite genes in three closely related marine Actinomycete species. Appl. Environ. Microbiol. 77, 7261–7270. 10.1128/AEM.05943-11 21873479 PMC3194868

[B15] Gallagher K. A. Fenical W. Jensen P. R. (2010). Hybrid isoprenoid secondary metabolite production in terrestrial and marine actinomycetes. Curr. Opin. Biotechnol. 21, 794–800. 10.1016/j.copbio.2010.09.010 20951024

[B16] Gallagher K. A. Rauscher K. Ioca L. P. Jensen P. R. (2013). Phylogenetic and chemical diversity of a hybrid-isoprenoidproducing *Streptomycete* lineage. Appl. Environ. Microbiol. 79, 6894–6902. 10.1128/AEM.01814-13 23995934 PMC3811536

[B17] Gill S. R. Fouts D. E. Archer G. L. Mongodin E. F. DeBoy R. T. Ravel J.. (2005). Insights on evolution of virulence and resistance from the complete genome analysis of an early methicillin-resistant *Staphylococcus aureus* strain and a biofilm-producing methicillin-resistant *Staphylococcus epidermidis* strain. J. Bacteriol. 187, 2426–2438. 10.1128/JB.187.7.2426-2438.2005 15774886 PMC1065214

[B18] Gontang E. A. Fenical W. Jensen P. R. (2007). Phylogenetic diversity of gram-positive bacteria cultured from marine sediments. Appl. Environ. Microbiol. 73, 3272–3282. 10.1128/AEM.02811-06 17400789 PMC1907118

[B19] Janssen P. H. Yates P. S. Grinton B. E. Taylor P. M. Sait M. (2002). Improved culturability of soil bacteria and isolation in pure culture of novel members of the divisions *Acidobacteria, Actinobacteria, Proteobacteria*, and *Verrucomicrobia*. Appl. Environ. Microbiol. 68, 2391–2396. 10.1128/AEM.68.5.2391-2396.2002 11976113 PMC127570

[B20] Jensen P. R. Gontang E. Mafnas C. Mincer T. J. Fenical W. (2005b). Culturable marine actinomycete diversity from tropical Pacific Ocean sediments. Environ. Microbiol. 7, 1039–1048. 10.1007/s10482-004-6540-1 15946301

[B21] Jensen P. R. Lauro F. M. (2008). An assessment of actinobacterial diversity in the marine environment. Antonie Van Leeuwenhoek 94, 51–62. 10.1007/s10482-008-9239-x 18500568 PMC3375478

[B22] Jensen P. R. Mafnas C. (2006). Biogeography of the marine actinomycete *Salinispora*. Environ. Microbiol. 8, 1881–1888. 10.1111/j.1462-2920.2006.01093.x 17014488

[B23] Jensen P. R. Mincer T. J. Williams P. G. Fenical W. (2005a). Marine actinomycete diversity and natural product discovery. Antonie Van Leeuwenhoek 87, 43–48. 10.1007/s10482-004-6540-1 15726290

[B24] Jensen P. R. Moore B. S. Fenical W. (2015). The marine actinomycete genus *Salinispora*: a model organism for secondary metabolite discovery. Nat. Prod. Rep. 32, 738–751. 10.1039/c4np00167b 25730728 PMC4414829

[B25] Lam K. S. (2006). Discovery of novel metabolites from marine actinomycetes. Curr. Opin. Microbiol. 9, 245–251. 10.1016/j.mib.2006.03.004 16675289

[B26] Lozupone C. Knight R. (2005). UniFrac: a new phylogenetic method for comparing microbial communities. Appl. Environ. Microbiol. 71, 8228–8235. 10.1128/AEM.71.12.8228-8235.2005 16332807 PMC1317376

[B27] Magarvey N. A. Keller J. M. Bernan V. Dworkin M. Sherman D. H. (2004). Isolation and characterization of novel marine-derived actinomycete taxa rich in bioactive metabolites. Appl. Environ. Microbiol. 70, 7520–7529. 10.1128/AEM.70.12.7520-7529.2004 15574955 PMC535209

[B28] Maldonado L. A. Fenical W. Jensen P. R. Kauffman C. A. Mincer T. J. Ward A. C.. (2005). *Salinispora arenicola* gen. nov., sp nov and *Salinispora tropica* sp nov., obligate marine actinomycetes belonging to the family *Micromonosporaceae*. Int. J. Syst. Evol. Microbiol. 55, 1759–1766. 10.1099/ijs.0.63625-0 16166663

[B29] Martiny A. C. Tai A. P. K. Veneziano D. Primeau F. Chisholm S. W. (2009). Taxonomic resolution, ecotypes and the biogeography of *Prochlorococcus*. Environ. Microbiol. 11, 823–832. 10.1111/j.1462-2920.2008.01803.x 19021692

[B30] Mato R. deLencastre H. Roberts R. B. Tomasz A. (1996). Multiplicity of genetic backgrounds among vancomycin-resistant *Enterococcus faecium* isolates recovered from an outbreak in a New York City Hospital. Microb. Drug Resist. 2, 309–317. 10.1089/mdr.1996.2.309 9158791

[B31] Nemergut D. R. Costello E. K. Hamady M. Lozupone C. Jiang L. Schmidt S. K.. (2011). Global patterns in the biogeography of bacterial taxa. Environ. Microbiol. 13, 135–144. 10.1111/j.1462-2920.2010.02315.x 21199253 PMC5834236

[B32] Pathom-aree W. Stach J. E. M. Ward A. C. Horikoshi K. Bull A. T. Goodfellow M. (2006). Diversity of actinomycetes isolated from Challenger Deep sediment (10,898 m) from the Mariana Trench. Extremophiles 10, 181–189. 10.1007/s00792-005-0482-z 16538400

[B33] Patin N. V. Duncan K. R. Dorrestein P. C. Jensen P. R. (2016). Competitive strategies differentiate closely related species of marine *Actinobacteria*. ISME J. 10, 478–490. 10.1038/ismej.2015.128 26241505 PMC4737938

[B34] Penn K. Jenkins C. Nett M. Udwary D. W. Gontang E. A. McGlinchey R. P.. (2009). Genomic islands link secondary metabolism to functional adaptation in marine *Actinobacteria*. ISME J. 3, 1193–1203. 10.1038/ismej.2009.58 19474814 PMC2749086

[B35] Prieto-Davó A. Villarreal-Gomez L. J. Forschner-Dancause S. Bull A. T. Stach J. E. Smith D. C.. (2013). Targeted search for actinomycetes from nearshore and deep-sea marine sediments. FEMS Microbiol. Ecol. 84, 510–518. 10.1111/1574-6941.12082 23360553 PMC3654085

[B36] Prudhomme J. McDaniel E. Ponts N. Bertani S. Fenical W. Jensen P.. (2008). Marine *Actinomycetes*: a new source of compounds against the human malaria parasite. PLoS ONE 3:e2335. 10.1371/journal.pone.0002335 18523554 PMC2391291

[B37] Rahman M. A. Islam M. Z. Islam M. A. U. (2011). Antibacterial activities of actinomycete isolates collected from soils of Rajshahi, Bangladesh. Biotechnol. Res. Int. 2011, 857925–857925. 10.4061/2011/857925 21904683 PMC3166718

[B38] Schloss P. D. Westcott S. L. Ryabin T. Hall J. R. Hartmann M. Hollister E. B.. (2009). Introducing mothur: open-source, platform-independent, community-supported software for describing and comparing microbial communities. Appl. Environ. Microbiol. 75, 7537–7541. 10.1128/AEM.01541-09 19801464 PMC2786419

[B39] Solano G. Rojas-Jiménez K. Jaspars M. Tamayo-Castillo G. (2009). Study of the diversity of culturable actinomycetes in the North Pacific and Caribbean coasts of Costa Rica. Antonie Van Leeuwenhoek 96, 71–78. 10.1007/s10482-009-9337-4 19365710 PMC3065112

[B40] Stach J. E. M. Maldonado L. A. Masson D. G. Ward A. C. Goodfellow M. Bull A. T. (2003). Statistical approaches for estimating actinobacterial diverity in marine sediments. Appl. Environ. Microbiol. 69, 6189–6200. 10.1128/AEM.69.10.6189-6200.2003 14532080 PMC201225

[B41] Subramani R. Aalbersberg W. (2012). Marine actinomycetes: an ongoing source of novel bioactive metabolites. Microbiol. Res. 167, 571–580. 10.1016/j.micres.2012.06.005 22796410

[B42] Takizawa M. Colwell R. R. Hill R. T. (1993). Isolation and diversity of actinomycetes in the Chesapeake Bay. Appl. Environ. Microbiol. 59, 997–1002. 16348922 10.1128/aem.59.4.997-1002.1993 PMC202228

[B43] Tamura K. Stecher G. Peterson D. Filipski A. Kumar S. (2013). MEGA6: molecular evolutionary genetics analysis version 6.0. Mol. Biol. Evol. 30, 2725–2729. 10.1093/molbev/mst197 24132122 PMC3840312

[B44] Tian X.-P. Xu Y. Zhang J. Li J. Chen Z. Kim C.-J.. (2012). *Streptomyces oceani* sp nov., a new obligate marine actinomycete isolated from a deep-sea sample of seep authigenic carbonate nodule in South China Sea. Antonie Van Leeuwenhoek 102, 335–343. 10.1007/s10482-012-9743-x 22696167

[B45] Vidgen M. E. Hooper J. N. A. Fuerst J. A. (2012). Diversity and distribution of the bioactive actinobacterial genus *Salinispora* from sponges along the Great Barrier Reef. Antonie Van Leeuwenhoek 101, 603–618. 10.1007/s10482-011-9676-9 22094709

[B46] Walker J. D. Colwell R. R. (1975). Factors affecting enumeration and isolation of actinomycetes from Chesapeake Bay and SouthEastern Atlantic Ocean sediments. Mar. Biol. 30, 193–201.

[B47] Weyland H. (1969). Actinomycetes in North Sea and Atlantic Ocean sediments. Nature 223, 858. 10.1038/223858a0 5799036

[B48] Weyland H. (1984). Actinomycetes of the bottom sediments of various seas, in 2 Deuxième Colloque International de Bactériologie Marine—CNRS (Brest: IFREMER), 73–79.

[B49] Williams D. E. Bernan V. S. Ritacco F. V. Maiese W. M. Greenstein M. Andersen R. J. (1999). Holyrines A and B, possible intermediates in staurosporine biosynthesis produced in culture by a marine actinomycete obtained from the North Atlantic Ocean. Tetrahedron Lett. 40, 7171–7174. 10.1016/S0040-4039(99)01495-1

