# Supplementary material for: The Madeira Archipelago As a Significant Source of Marine-Derived Actinomycete Diversity with Anticancer and Antimicrobial Potential
Source: Front Microbiol. 2016 Oct 7;7:1594. doi: 10.3389/fmicb.2016.01594 (PMC5053986; doi:10.3389/fmicb.2016.01594)
Supplement: Supplementary file 1 [file Table1.docx]

| **PTM**  **Supplementary Table 1**. List of all strains isolated from the Madeira Archipelago including their habitat and accession number from GenBank (AccessID). | **Habitat** | **AccesID** |
| --- | --- | --- |
| PTM-264 | deep sand edge of rocky slope | KT446004 |
| PTM-362 | deep sand edge of rocky slope | KT446007 |
| PTM-385 | deep sand edge of rocky slope | KT446008 |
| PTM-382 | deep sand edge of rocky slope | KT446011 |
| PTM-197 | deep sand edge of rocky slope | KT446017 |
| PTM-64 | deep sand edge of rocky slope | KT446018 |
| PTM-322 | deep sand edge of rocky slope | KT446021 |
| PTM-338 | deep sand edge of rocky slope | KT446032 |
| PTM-119 | deep sand edge of rocky slope | KT446033 |
| PTM-210 | deep sand edge of rocky slope | KT446037 |
| PTM-270 | deep sand edge of rocky slope | KT446040 |
| PTM-262 | deep sand edge of rocky slope | KT446047 |
| PTM-53 | deep sand edge of rocky slope | KT446048 |
| PTM-260 | deep sand edge of rocky slope | KT446049 |
| PTM-136 | deep sand edge of rocky slope | KT446050 |
| PTM-364 | deep sand edge of rocky slope | KT446082 |
| PTM-182 | deep sand edge of rocky slope | KT446084 |
| PTM-191 | deep sand edge of rocky slope | KT446093 |
| PTM-334 | deep sand edge of rocky slope | KT446097 |
| PTM-61 | deep sand edge of rocky slope | KT446098 |
| PTM-300 | deep sand edge of rocky slope | KT446108 |
| PTM-368 | deep sand edge of rocky slope | KT446113 |
| PTM-374 | deep sand edge of rocky slope | KT446114 |
| PTM-352 | deep sand edge of rocky slope | KT446116 |
| PTM-406 | deep sand edge of rocky slope | KT446133 |
| PTM-341 | deep sand edge of rocky slope | KT446134 |
| PTM-154 | deep sand edge of rocky slope | KT446136 |
| PTM-51 | deep sand edge of rocky slope | KT446140 |
| PTM-416 | deep sand edge of rocky slope | KT446151 |
| PTM-69 | deep sand edge of rocky slope | KT446152 |
| PTM-344 | deep sand edge of rocky slope | KT446154 |
| PTM-250 | deep sand edge of rocky slope | KT446156 |
| PTM-394 | deep sand edge of rocky slope | KT446161 |
| PTM-394 | deep sand edge of rocky slope | KT446161 |
| PTM-65 | deep sand edge of rocky slope | KT446163 |
| PTM-186 | deep sand edge of rocky slope | KT446167 |
| PTM-299 | deep sand edge of rocky slope | KT446171 |
| PTM-198 | deep sand edge of rocky slope | KT446188 |
| PTM-129 | deep sand edge of rocky slope | KT446196 |
| PTM-162 | deep sand edge of rocky slope | KT446204 |
| PTM-27 | deep sand edge of rocky slope | KT446205 |
| PTM-112 | deep sand edge of rocky slope | KT446207 |
| PTM-92 | deep sand edge of rocky slope | KT446210 |
| PTM-169 | deep sand edge of rocky slope | KT446213 |
| PTM-358 | deep sand edge of rocky slope | KT446214 |
| **PTM** | **Habitat** | **AccesID** |
| PTM-244 | deep sand edge of rocky slope | KT446217 |
| PTM-290 | deep sand edge of rocky slope | KT446229 |
| PTM-227 | deep sand edge of rocky slope | KT446234 |
| PTM-310 | deep sand edge of rocky slope | KT446241 |
| PTM-114 | deep sand edge of rocky slope | KT446251 |
| PTM-215 | deep sand edge of rocky slope | KT446253 |
| PTM-204 | deep sand edge of rocky slope | KT446254 |
| PTM-08 | deep sand edge of rocky slope | KT446256 |
| PTM-02 | deep sand edge of rocky slope | KT446260 |
| PTM-206 | deep sand edge of rocky slope | KT446261 |
| PTM-229 | deep sand edge of rocky slope | KT446266 |
| PTM-419 | deep sand edge of rocky slope | KT446267 |
| PTM-408 | deep sand edge of rocky slope | KT446268 |
| PTM-09 | deep sand edge of rocky slope | KT446272 |
| PTM-166 | deep sand edge of rocky slope | KT446273 |
| PTM-131 | deep sand edge of rocky slope | KT446276 |
| PTM-175 | deep sand edge of rocky slope | KT446277 |
| PTM-212 | deep sand edge of rocky slope | KT446278 |
| PTM-183 | deep sand edge of rocky slope | KT446282 |
| PTM-280 | deep sand edge of rocky slope | KT446283 |
| PTM-328 | deep sand edge of rocky slope | KT446284 |
| PTM-47 | deep sand edge of rocky slope | KT446285 |
| PTM-72 | deep sand edge of rocky slope | KT446287 |
| PTM-205 | deep sand edge of rocky slope | KT446289 |
| PTM-109 | deep sand edge of rocky slope | KT446290 |
| PTM-216 | deep sand edge of rocky slope | KT446292 |
| PTM-384 | deep sand edge of rocky slope | KT446295 |
| PTM-329 | deep sand edge of rocky slope | KT446296 |
| PTM-163 | deep sand edge of rocky slope | KT446298 |
| PTM-272 | deep sand edge of rocky slope | KT446305 |
| PTM-315 | deep sand edge of rocky slope | KT446310 |
| PTM-367 | deep sand edge of rocky slope | KT446320 |
| PTM-327 | deep sand edge of rocky slope | KT446321 |
| PTM-222 | deep sand edge of rocky slope | KT446323 |
| PTM-284 | deep sand edge of rocky slope | KT446324 |
| PTM-226 | deep sand edge of rocky slope | KT446326 |
| PTM-283 | deep sand edge of rocky slope | KT446328 |
| PTM-187 | deep sand edge of rocky slope | KT446335 |
| PTM-180 | deep sand edge of rocky slope | KT446338 |
| PTM-349 | deep sand edge of rocky slope | KT446343 |
| PTM-220 | deep sand edge of rocky slope | KT446344 |
| PTM-168 | deep sand edge of rocky slope | KT446347 |
| PTM-146 | deep sand edge of rocky slope | KT446348 |
| PTM-213 | deep sand edge of rocky slope | KT446349 |
| PTM-359 | deep sand edge of rocky slope | KT446350 |
| **PTM** | **HABITAT** | **ACCESSID** |
| PTM-307 | deep sand edge of rocky slope | KT446351 |
| PTM-199 | deep sand edge of rocky slope | KT446352 |
| PTM-200 | deep sand edge of rocky slope | KT446353 |
| PTM-108 | deep sand edge of rocky slope | KT446354 |
| PTM-211 | deep sand edge of rocky slope | KT446364 |
| PTM-209 | deep sand edge of rocky slope | KT446371 |
| PTM-71 | deep sand edge of rocky slope | KT446376 |
| PTM-318 | deep sand edge of rocky slope | KT446379 |
| PTM-50 | deep sand edge of rocky slope | KT446382 |
| PTM-256 | deep sand edge of rocky slope | KT446385 |
| PTM-312 | deep sand edge of rocky slope | KT446386 |
| PTM-218 | deep sand edge of rocky slope | KT446391 |
| PTM-361 | deep sand edge of rocky slope | KT446392 |
| PTM-421 | deep sand edge of rocky slope | KT446394 |
| PTM-231 | deep sand edge of rocky slope | KT446395 |
| PTM-289 | off rocky shore | KT446121 |
| PTM-184 | off rocky shore | KT446123 |
| PTM-156 | off rocky shore | KT446124 |
| PTM-127 | off rocky shore | KT446125 |
| PTM-34 | off rocky shore | KT446147 |
| PTM-313 | off rocky shore | KT446184 |
| PTM-177 | off rocky shore | KT446190 |
| PTM-232 | off rocky shore | KT446223 |
| PTM-24 | off rocky shore | KT446224 |
| PTM-26 | off rocky shore | KT446225 |
| PTM-217 | off rocky shore | KT446226 |
| PTM-377 | off rocky shore | KT446245 |
| PTM-221 | off rocky shore | KT446257 |
| PTM-164 | off rocky shore | KT446263 |
| PTM-173 | off rocky shore | KT446306 |
| PTM-230 | off rocky shore | KT446322 |
| PTM-288 | off rocky shore | KT446325 |
| PTM-170 | off rocky shore | KT446345 |
| PTM-354 | off rocky shore | KT446368 |
| PTM-370 | off rocky shore | KT446389 |
| PTM-386 | Off sandy beach | KT446010 |
| PTM-411 | Off sandy beach | KT446038 |
| PTM-13 | Off sandy beach | KT446293 |
| PTM-11 | Off sandy beach | KT446294 |
| PTM-202 | Off sandy beach | KT446317 |
| PTM-176 | Off sandy beach | KT446329 |
| PTM-239 | Off sandy beach | KT446342 |
| PTM-30 | off shore | KT446005 |
| PTM-37 | off shore | KT446149 |
| PTM-178 | off shore | KT446165 |
| **PTM** | **HABITAT** | **ACCESSID** |
| PTM-201 | off shore | KT446258 |
| PTM-271 | off shore | KT446259 |
| PTM-107 | off shore | KT446279 |
| PTM-28 | off shore | KT446286 |
| PTM-224 | off shore | KT446291 |
| PTM-324 | off shore | KT446330 |
| PTM-298 | off shore | KT446377 |
| PTM-326 | off shore | KT446388 |
| PTM-70 | offshore | KT446006 |
| PTM-194 | offshore | KT446013 |
| PTM-132 | offshore | KT446014 |
| PTM-185 | offshore | KT446015 |
| PTM-248 | offshore | KT446016 |
| PTM-137 | offshore | KT446023 |
| PTM-363 | offshore | KT446024 |
| PTM-77 | offshore | KT446025 |
| PTM-309 | offshore | KT446026 |
| PTM-87 | offshore | KT446041 |
| PTM-35 | offshore | KT446046 |
| PTM-110 | offshore | KT446057 |
| PTM-17 | offshore | KT446144 |
| PTM-297 | offshore | KT446150 |
| PTM-257 | offshore | KT446155 |
| PTM-23 | offshore | KT446157 |
| PTM-82 | offshore | KT446158 |
| PTM-223 | offshore | KT446175 |
| PTM-29 | offshore | KT446192 |
| PTM-398 | offshore | KT446193 |
| PTM-282 | offshore | KT446255 |
| PTM-62 | offshore | KT446299 |
| PTM-203 | offshore | KT446308 |
| PTM-161 | offshore | KT446312 |
| PTM-20 | offshore | KT446314 |
| PTM-214 | offshore | KT446315 |
| PTM-95 | offshore | KT446336 |
| PTM-94 | offshore | KT446340 |
| PTM-79 | offshore | KT446367 |
| PTM-301 | offshore | KT446369 |
| PTM-12 | offshore | KT446372 |
| PTM-43 | offshore | KT446375 |
| PTM-303 | offshore | KT446390 |
| PTM-104 | rocky shoreline | KT446002 |
| PTM-286 | rocky shoreline | KT446003 |
| PTM-316 | rocky shoreline | KT446020 |
| PTM-153 | rocky shoreline | KT446027 |
| **PTM** | **HABITAT** | **ACCESSID** |
| PTM-22 | rocky shoreline | KT446029 |
| PTM-135 | rocky shoreline | KT446030 |
| PTM-372 | rocky shoreline | KT446034 |
| PTM-148 | rocky shoreline | KT446039 |
| PTM-151 | rocky shoreline | KT446051 |
| PTM-155 | rocky shoreline | KT446052 |
| PTM-196 | rocky shoreline | KT446053 |
| PTM-252 | rocky shoreline | KT446054 |
| PTM-278 | rocky shoreline | KT446056 |
| PTM-140 | rocky shoreline | KT446058 |
| PTM-90 | rocky shoreline | KT446059 |
| PTM-150 | rocky shoreline | KT446060 |
| PTM-75 | rocky shoreline | KT446062 |
| PTM-390 | rocky shoreline | KT446063 |
| PTM-139 | rocky shoreline | KT446064 |
| PTM-36 | rocky shoreline | KT446065 |
| PTM-355 | rocky shoreline | KT446067 |
| PTM-179 | rocky shoreline | KT446068 |
| PTM-120 | rocky shoreline | KT446069 |
| PTM-392 | rocky shoreline | KT446070 |
| PTM-366 | rocky shoreline | KT446072 |
| PTM-159 | rocky shoreline | KT446073 |
| PTM-357 | rocky shoreline | KT446074 |
| PTM-304 | rocky shoreline | KT446075 |
| PTM-302 | rocky shoreline | KT446076 |
| PTM-145 | rocky shoreline | KT446077 |
| PTM-144 | rocky shoreline | KT446078 |
| PTM-246 | rocky shoreline | KT446079 |
| PTM-171 | rocky shoreline | KT446080 |
| PTM-106 | rocky shoreline | KT446085 |
| PTM-15 | rocky shoreline | KT446086 |
| PTM-149 | rocky shoreline | KT446089 |
| PTM-376 | rocky shoreline | KT446090 |
| PTM-331 | rocky shoreline | KT446091 |
| PTM-274 | rocky shoreline | KT446092 |
| PTM-21 | rocky shoreline | KT446094 |
| PTM-01 | rocky shoreline | KT446095 |
| PTM-134 | rocky shoreline | KT446096 |
| PTM-125 | rocky shoreline | KT446099 |
| PTM-133 | rocky shoreline | KT446100 |
| PTM-76 | rocky shoreline | KT446101 |
| PTM-152 | rocky shoreline | KT446102 |
| PTM-251 | rocky shoreline | KT446103 |
| PTM-261 | rocky shoreline | KT446104 |
| PTM-295 | rocky shoreline | KT446105 |
| **PTM** | **HABITAT** | **ACCESSID** |
| PTM-243 | rocky shoreline | KT446106 |
| PTM-330 | rocky shoreline | KT446107 |
| PTM-03 | rocky shoreline | KT446110 |
| PTM-395 | rocky shoreline | KT446112 |
| PTM-241 | rocky shoreline | KT446115 |
| PTM-10 | rocky shoreline | KT446117 |
| PTM-46 | rocky shoreline | KT446118 |
| PTM-124 | rocky shoreline | KT446119 |
| PTM-335 | rocky shoreline | KT446120 |
| PTM-63 | rocky shoreline | KT446126 |
| PTM-85 | rocky shoreline | KT446127 |
| PTM-126 | rocky shoreline | KT446128 |
| PTM-80 | rocky shoreline | KT446129 |
| PTM-255 | rocky shoreline | KT446130 |
| PTM-57 | rocky shoreline | KT446131 |
| PTM-143 | rocky shoreline | KT446132 |
| PTM-58 | rocky shoreline | KT446135 |
| PTM-294 | rocky shoreline | KT446137 |
| PTM-259 | rocky shoreline | KT446141 |
| PTM-39 | rocky shoreline | KT446142 |
| PTM-158 | rocky shoreline | KT446143 |
| PTM-138 | rocky shoreline | KT446145 |
| PTM-265 | rocky shoreline | KT446186 |
| PTM-339 | rocky shoreline | KT446187 |
| PTM-66 | rocky shoreline | KT446189 |
| PTM-420 | rocky shoreline | KT446194 |
| PTM-247 | rocky shoreline | KT446197 |
| PTM-375 | rocky shoreline | KT446199 |
| PTM-245 | rocky shoreline | KT446201 |
| PTM-141 | rocky shoreline | KT446202 |
| PTM-279 | rocky shoreline | KT446203 |
| PTM-249 | rocky shoreline | KT446206 |
| PTM-157 | rocky shoreline | KT446208 |
| PTM-56 | rocky shoreline | KT446211 |
| PTM-99 | rocky shoreline | KT446218 |
| PTM-397 | rocky shoreline | KT446227 |
| PTM-311 | rocky shoreline | KT446228 |
| PTM-68 | rocky shoreline | KT446231 |
| PTM-356 | rocky shoreline | KT446232 |
| PTM-369 | rocky shoreline | KT446233 |
| PTM-96 | rocky shoreline | KT446236 |
| PTM-115 | rocky shoreline | KT446238 |
| PTM-98 | rocky shoreline | KT446240 |
| PTM-291 | rocky shoreline | KT446242 |
| PTM-325 | rocky shoreline | KT446246 |
| **PTM** | **HABITAT** | **ACCESSID** |
| PTM-235 | rocky shoreline | KT446248 |
| PTM-323 | rocky shoreline | KT446249 |
| PTM-207 | rocky shoreline | KT446271 |
| PTM-273 | rocky shoreline | KT446301 |
| PTM-336 | rocky shoreline | KT446313 |
| PTM-317 | rocky shoreline | KT446316 |
| PTM-319 | rocky shoreline | KT446331 |
| PTM-32 | rocky shoreline | KT446334 |
| PTM-285 | rocky shoreline | KT446356 |
| PTM-381 | rocky shoreline | KT446358 |
| PTM-276 | rocky shoreline | KT446359 |
| PTM-348 | rocky shoreline | KT446366 |
| PTM-320 | rocky shoreline | KT446370 |
| PTM-399 | rocky shoreline | KT446373 |
| PTM-31 | rocky shoreline | KT446374 |
| PTM-388 | rocky shoreline | KT446378 |
| PTM-351 | rocky shoreline | KT446380 |
| PTM-389 | rocky shoreline | KT446381 |
| PTM-383 | rocky shoreline | KT446383 |
| PTM-410 | rocky shoreline | KT446384 |
| PTM-228 | rocky shoreline | KT446387 |
| PTM-387 | rocky shoreline | KT446397 |
| PTM-353 | rocky shoreline | KT446399 |
| PTM-401 | Rocky shoreline, sand patches | KT446009 |
| PTM-321 | Rocky shoreline, sand patches | KT446022 |
| PTM-267 | Rocky shoreline, sand patches | KT446031 |
| PTM-111 | Rocky shoreline, sand patches | KT446042 |
| PTM-409 | Rocky shoreline, sand patches | KT446043 |
| PTM-83 | Rocky shoreline, sand patches | KT446044 |
| PTM-100 | Rocky shoreline, sand patches | KT446071 |
| PTM-371 | Rocky shoreline, sand patches | KT446083 |
| PTM-373 | Rocky shoreline, sand patches | KT446087 |
| PTM-147 | Rocky shoreline, sand patches | KT446109 |
| PTM-167 | Rocky shoreline, sand patches | KT446176 |
| PTM-340 | Rocky shoreline, sand patches | KT446212 |
| PTM-293 | Rocky shoreline, sand patches | KT446215 |
| PTM-91 | Rocky shoreline, sand patches | KT446216 |
| PTM-93 | Rocky shoreline, sand patches | KT446219 |
| PTM-25 | Rocky shoreline, sand patches | KT446220 |
| PTM-44 | Rocky shoreline, sand patches | KT446221 |
| PTM-240 | Rocky shoreline, sand patches | KT446222 |
| PTM-78 | Rocky shoreline, sand patches | KT446235 |
| PTM-233 | Rocky shoreline, sand patches | KT446237 |
| PTM-89 | Rocky shoreline, sand patches | KT446239 |
| PTM-380 | Rocky shoreline, sand patches | KT446244 |
| **PTM** | **HABITAT** | **ACCESSID** |
| PTM-60 | Rocky shoreline, sand patches | KT446247 |
| PTM-305 | Rocky shoreline, sand patches | KT446250 |
| PTM-172 | Rocky shoreline, sand patches | KT446262 |
| PTM-160 | Rocky shoreline, sand patches | KT446269 |
| PTM-174 | Rocky shoreline, sand patches | KT446274 |
| PTM-14 | Rocky shoreline, sand patches | KT446300 |
| PTM-16 | Rocky shoreline, sand patches | KT446302 |
| PTM-105 | Rocky shoreline, sand patches | KT446303 |
| PTM-101 | Rocky shoreline, sand patches | KT446327 |
| PTM-113 | Rocky shoreline, sand patches | KT446333 |
| PTM-102 | Rocky shoreline, sand patches | KT446337 |
| PTM-342 | Rocky shoreline, sand patches | KT446339 |
| PTM-54 | Rocky shoreline, sand patches | KT446341 |
| PTM-292 | Rocky shoreline, sand patches | KT446357 |
| PTM-88 | Rocky shoreline, sand patches | KT446360 |
| PTM-84 | Rocky shoreline, sand patches | KT446361 |
| PTM-237 | Rocky shoreline, sand patches | KT446365 |
| PTM-40 | rocky with lots of Diadema | KT446035 |
| PTM-116 | rocky with lots of Diadema | KT446045 |
| PTM-415 | rocky with lots of Diadema | KT446061 |
| PTM-181 | rocky with lots of Diadema | KT446111 |
| PTM-42 | rocky with lots of Diadema | KT446148 |
| PTM-06 | rocky with lots of Diadema | KT446159 |
| PTM-332 | rocky with lots of Diadema | KT446162 |
| PTM-55 | rocky with lots of Diadema | KT446252 |
| PTM-306 | rocky with lots of Diadema | KT446265 |
| PTM-73 | rocky with lots of Diadema | KT446275 |
| PTM-238 | rocky with lots of Diadema | KT446297 |
| PTM-04 | rocky with lots of Diadema | KT446304 |
| PTM-236 | rocky with lots of Diadema | KT446309 |
| PTM-195 | rocky with lots of Diadema | KT446355 |
| PTM-165 | sand bottom | KT446146 |
| PTM-18 | sand bottom | KT446168 |
| PTM-86 | sand bottom | KT446170 |
| PTM-337 | sand bottom | KT446185 |
| PTM-122 | sand bottom | KT446200 |
| PTM-242 | sand bottom | KT446209 |
| PTM-365 | sand bottom | KT446230 |
| PTM-360 | sand bottom | KT446243 |
| PTM-225 | sand bottom | KT446270 |
| PTM-49 | sand bottom | KT446307 |
| PTM-48 | sand bottom | KT446311 |
| PTM-350 | sand bottom | KT446318 |
| PTM-343 | sand bottom | KT446363 |
| PTM-103 | sand edge of rocky shoreline | KT446036 |
| **PTM** | **HABITAT** | **ACCESSID** |
| PTM-333 | sand edge of rocky shoreline | KT446055 |
| PTM-268 | sand edge of rocky shoreline | KT446081 |
| PTM-417 | sand edge of rocky shoreline | KT446160 |
| PTM-121 | sand edge of rocky shoreline | KT446180 |
| PTM-404 | sand edge of rocky shoreline | KT446181 |
| PTM-81 | sand edge of rocky shoreline | KT446195 |
| PTM-188 | sand edge of rocky shoreline | KT446198 |
| PTM-234 | sand edge of rocky shoreline | KT446319 |
| PTM-393 | sand edge of rocky shoreline | KT446332 |
| PTM-418 | sand edge of rocky shoreline | KT446393 |
| PTM-67 | sand edge of rocky shoreline | KT446398 |
| PTM-405 | sand edge of rocky shoreline | KT446401 |
| PTM-414 | sand | KT446012 |
| PTM-258 | sand | KT446019 |
| PTM-254 | sand | KT446028 |
| PTM-189 | sand | KT446066 |
| PTM-266 | sand | KT446088 |
| PTM-07 | sand | KT446122 |
| PTM-123 | sand | KT446138 |
| PTM-314 | sand | KT446139 |
| PTM-296 | sand | KT446164 |
| PTM-281 | sand | KT446166 |
| PTM-263 | sand | KT446169 |
| PTM-413 | sand | KT446172 |
| PTM-118 | sand | KT446173 |
| PTM-190 | sand | KT446174 |
| PTM-19 | Sand | KT446177 |
| PTM-05 | sand | KT446178 |
| PTM-412 | sand | KT446179 |
| PTM-396 | sand | KT446182 |
| PTM-403 | sand | KT446183 |
| PTM-346 | sand | KT446191 |
| PTM-400 | sand | KT446264 |
| PTM-45 | sand | KT446280 |
| PTM-193 | sand | KT446281 |
| PTM-219 | sand | KT446288 |
| PTM-275 | sand | KT446346 |
| PTM-402 | sand | KT446362 |
| PTM-379 | sand | KT446396 |
| PTM-41 | sand | KT446400 |
